# The Safety, Acceptability, and Feasibility of Single-Dose Rifampicin as Post-Exposure Chemoprophylaxis for Contacts of Leprosy Patients in Togo: A Mixed-Method Sequential Explanatory Study

**DOI:** 10.3390/tropicalmed9110276

**Published:** 2024-11-14

**Authors:** Akila Wimima Bakoubayi, Falapalaki Haliba, Wendpouiré Ida C. Zida-Compaore, P’tanam P’kontème Bando, Yao Rodion Konu, Abissouwèssim Egbare Tchade, Kodjo Akpadja, Kamevor Alaglo, Maweke Tchalim, P’niwè Patchali, Yaovi Djakpa, Komi Amekuse, Piham Gnossike, Denis A. Yawovi Gadah, Christa Kasang, Didier Koumavi Ekouevi

**Affiliations:** 1 German Leprosy and Tuberculosis Relief Association, Lome 2271, Togo; jeremy.haliba@dahw-global.org (F.H.); brice.bando@dahw-global.org (P.P.B.); yawovi.djakpa@dahw-global.org (Y.D.); komi.amekuse@dahw-global.org (K.A.); denis.gadah@dahw-global.org (D.A.Y.G.); 2 Department of Public Health, Faculty of Health Sciences, University of Lome, Lome 1515, Togo; zidaidacarine@gmail.com (W.I.C.Z.-C.); rodionko@yahoo.fr (Y.R.K.); boutelezitchade@gmail.com (A.E.T.); didier.ekouevi@gmail.com (D.K.E.); 3 National Tuberculosis Control Program, Lome 2271, Togo; akpadjakodjo2018@gmail.com; 4 National Neglected Tropical Diseases Control Program, Lome 336, Togo; alaglokamevor2014@gmail.com (K.A.); tmaweke@yahoo.fr (M.T.); patchalijudith@gmail.com (P.P.); jacquesgnoss@yahoo.fr (P.G.); 5 German Leprosy and Tuberculosis Relief Association, 97080 Würzburg, Germany; dr.christa.kasang@dahw.de

**Keywords:** leprosy, single-dose rifampicin, post-exposure prophylaxis, acceptability, feasibility, Togo

## Abstract

The World Health Organization is encouraging countries to include contact screening and single-dose rifampicin administration as preventive chemotherapy for contacts of leprosy patients in their leprosy control activities. However, no study has been conducted to assess the safety of SDR-PEP and the acceptability and feasibility of this intervention in Togo. To assess the safety of SDR-PEP, we used a cohort design, and for acceptability and feasibility, we used a mixed method, combining a quantitative study to assess the safety of SDR-PEP in a cohort of contacts from recently diagnosed leprosy patients followed by a qualitative study to identify the social, cultural, or institutional factors that would influence the adoption of single-dose rifampicin as post-exposure prophylaxis for contacts of leprosy patients in Togo. For the quantitative study, all identified index patients agreed to the disclosure of their status to their contacts and provided a list of their contacts. All the contacts found agreed to take part in the study, and an appointment was made for screening. However, some contacts were absent on the screening day for no reason. All eligible contacts agreed to take SDR and were followed up after taking the drug. No severe adverse events were reported during the follow-up. For the qualitative study, 72 interviews (66 semi-structured interviews and 6 focus groups) were carried out, and it emerged that, overall, opinions were favorable on the acceptability and feasibility of implementing single-dose rifampicin as post-exposure prophylaxis for contacts of leprosy patients in Togo. However, a number of conditions need to be considered for more effective results.

## 1. Introduction

Chemoprophylaxis is defined as the administration of drugs capable of preventing infection or preventing infected people from becoming ill [1,2]. In the case of leprosy control, this means giving medicines to people in contact with leprosy patients and, therefore, potentially infected with Mycobacterium leprae to prevent them from becoming ill [3].

Numerous studies have been carried out to identify suitable post-exposure prophylaxis for leprosy. The first studies on chemoprophylaxis for contacts of leprosy patients focused on dapsone and acedapsone in the 1960s and 1970s and rifampicin in the 1980s and 1990s [4].

In 1988, single-dose rifampicin as post-exposure prophylaxis (SDR-PEP) was first studied in the South Marquesas Islands in an uncontrolled trial [1,2]. A follow-up survey ten years later showed that the efficacy of chemoprophylaxis varied between 35 and 45% [5]. A randomized double-blind controlled trial, COLEP, carried out in Bangladesh between 2002 and 2007, reported an overall reduction in leprosy risk of 57% among contacts of leprosy patients [6,7]. In view of these results, the World Health Organization (WHO) recommended in 2018 contact screening and single-dose rifampicin (SDR) administration as preventive chemotherapy for contacts of leprosy patients to be included in routine leprosy control activities [8].

In Togo, West Africa, a study carried out in 2014 revealed the carriage of viable mycobacteria in contacts with an average of 10 contacts per index patient, some of whom subsequently developed the disease [9]. The number of new cases has risen steadily over the past five years. In 2021, 132 new leprosy cases (1.6 cases per 100,000 inhabitants) were recorded, compared with 67 cases (1 case per 100,000 inhabitants) in 2018 [10]. These results show the persistence of leprosy and the need to take contact cases into account in leprosy control strategies in Togo. Although contact screening and the administration of SDR as preventive chemotherapy for contacts of leprosy patients have been recommended by the WHO and already deployed in some African countries, to our knowledge, no studies have been conducted to assess the safety of this intervention in West Africa and the acceptability and feasibility of implementation in Togo. Evidence at the national level on the acceptability and feasibility of contact tracing and SDR administration in routine leprosy control activities is needed. The aim of the study was to assess the safety, acceptability, and feasibility of implementing SDR-PEP for contacts of leprosy patients in Togo.

## 2. Methods

A mixed-method sequential explanatory study was carried out, combining a quantitative study followed by a qualitative study.

### 2.1. Quantitative Study

#### 2.1.1. Type, Setting, Study Population, and Period of Study

**Figure 1:**
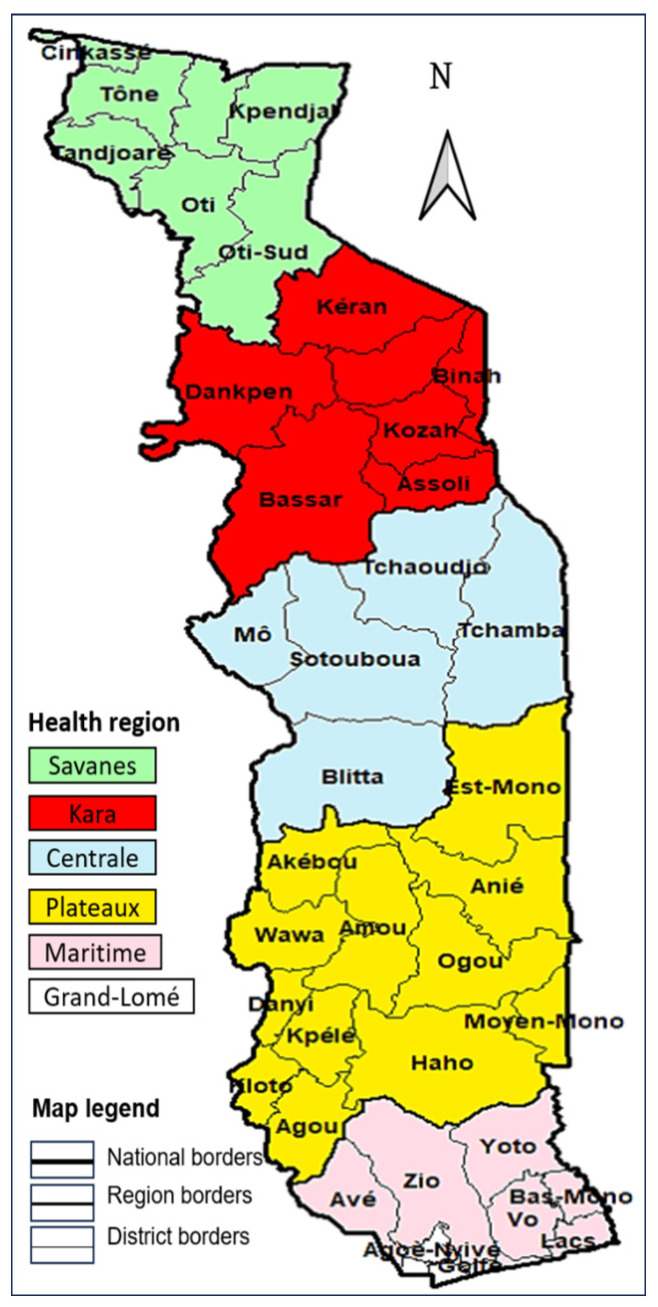
Togo regions and health districts.

The quantitative study is a longitudinal cohort study of contacts of leprosy patients identified in 2021 in the Maritime region. Togo has six health regions and 39 health districts (Figure 1). Contacts were recruited between 3 and 29 March 2022 and followed up for a period of 10 months.

Subjects included in the study were as follows: (i) identified as contacts of a leprosy patient; (ii) residing in the Maritime region; (iii) having given their consent to participate in the study; (iv) over 5 years of age.

Subjects not included in the study were as follows: (i) with possible signs and/or symptoms of leprosy or tuberculosis; (ii) with a history of liver or kidney disorders; (iii) pregnant women; (iv) who had received rifampicin in the last two years; (v) with a history of allergy to rifampicin.

#### 2.1.2. Operational Definitions

Index case: any person diagnosed with leprosy for the first time.

Contact: Any person who had been in contact with an untreated case of leprosy for at least 20 h per week for at least three months during the past year was considered a contact [11]. Three types of contact were distinguished [11]:

✓ Household contact: a person living in the same household as an index case or sharing the same meal with him/her.
✓ Neighbor contact: a person living in the immediate vicinity of an index case or in a neighboring household less than 100 m away.
✓ Social contact: any other person who has been in prolonged contact with an index case and who is not classified as a family or neighbor contact (friends or people sharing a workplace or attending the same school or leisure area).

Two clinical forms of leprosy are distinguished:

✓ Pauci-bacillary: five or fewer lesions with no bacteria detected in the skin smear (sample taken from the area).
✓ Multi-bacillary: more than five lesions or the detection of one or both bacteria in the skin smear.

#### 2.1.3. Study Process

The study was carried out in 4 phases:

✓ Identification and enrolment of index cases

Index cases were leprosy patients detected in 2021 in the Maritime region. Using information provided by the patients at the time of diagnosis, trained community health workers visited the patients to present the study and obtain their consent to participate. They thus consented to give the list of their contacts and to divulge their status to their contacts.

✓ Contact identification and enrolment

The community health workers visited the homes of identified contacts to introduce the study and provide information on the safety, side effects, and usefulness of prophylaxis, in order to obtain their consent to participate in the study.

✓ Screening and administration of SDR to contacts

Once consent had been obtained, contacts were invited on a given day to a health facility in their community or an appointment was made at their home or at a location indicated according to their preference, for screening prior to the administration of rifampicin. Screening consisted of examining contacts for signs and symptoms of leprosy by whole-body skin screening (with the aim of detecting leprosy), for other conditions (e.g., tuberculosis), or for other non-inclusion criteria. Contacts meeting the inclusion criteria received SDR based on their age and/or weight according to WHO guidelines [8]. People aged 15 and over received a single dose of 600 mg, people aged 9 to 14 received a single dose of 450 mg, children aged 6 to below 9 or 8 years weighing ≥ 20 kg received a single dose of 300 mg, and those weighing < 20 kg received a single dose of 150 mg.

✓ Contact follow-up

Contacts who received rifampicin were followed by community health workers who visited their homes every day for the first week, once a week for the first month, and once a month for ten months to collect any adverse events. When an adverse event occurred, the community health worker informed the study implementation team, who analyzed it to look for a causal relationship between the adverse event and rifampicin before concluding whether or not it was due to the dose of rifampicin taken. However, all adverse events reported as being related or unrelated to the use of rifampicin were treated free of charge by the research team. All information relating to adverse events was recorded on specially prepared contact follow-up report forms.

#### 2.1.4. Data Collection

A questionnaire was used to collect socio-demographic (age, sex, level of education, marital status, profession, area of residence, etc.) and clinical information on patients with leprosy.

For contacts, data were collected at the time of inclusion, and they included socio-demographic characteristics (age, sex, level of education, marital status, profession, area of residence, etc.), category of contact (family, neighbor, social), screening result (signs of leprosy or tuberculosis), and other SDR administration criteria. During follow-up, information on adverse events and their management was documented.

During the implementation of the strategy, the number of leprosy patients who have agreed to disclose their status to their contacts and have agreed to give a list of their contacts, as well as the number of identified contacts who have agreed to be screened and to take SDR, will be considered in assessing the acceptability. The number of contacts traced by community health workers, the number of contacts screened, and the number of contacts who received SDR and were followed up will serve as criteria for assessing the intervention’s feasibility.

To study the acceptability of the strategy, the acceptability of index cases making their status known to contacts and giving a list of their contacts (family, social, and neighborhood contacts) and the acceptability of contacts being screened and taking rifampicin were assessed. Contact tracing by the community health worker, screening and administration of SDR to contacts, and follow-up of contacts for documentation and management of adverse events were studied to assess the feasibility of the strategy.

#### 2.1.5. Data Processing and Analysis

Data were collected in individual observation notebooks for each index patient and his contacts, entered into an Epidata 3.1 database, and exported to R software version 4.3.0 for analysis. The results are presented as proportions for qualitative variables and means and medians for quantitative variables.

### 2.2. Qualitative Study

#### 2.2.1. Setting and Period of Study

The study was carried out from 22 May 2023 to 19 June 2023 in all health regions of Togo (Figure 1). Semi-structured interviews and focus groups were conducted.

#### 2.2.2. Study Population

The population consisted of the main actors in the implementation of health interventions of the national neglected tropical diseases program, health directors, and leprosy patients and their contacts.

#### 2.2.3. Sampling

Rational sampling was used within each defined target group, balanced according to socio-cultural context, geographical distribution, and gender. Thus, semi-structured interviews were conducted with 12 index patients (6 men and 6 women; 2 per region), 12 contacts (6 men and 6 women; 2 per region), 12 community health workers (6 men and 6 women; 2 per region), 12 community leaders (6 men and 6 women; 2 per region), 3 regional neglected tropical disease focal points, 6 district regional neglected tropical disease focal points, 6 health directors (2 regional health directors and 4 health district directors), and 3 members of the national neglected tropical diseases program.

Focus groups with 4 to 8 participants were conducted in two cities, one in Kara (in the north of the country) and the other in Tsévié (in the south of the country), with 1 group of index patients (men), 1 group of index patients (women), 1 contact group (men), 1 contact group (women), and 2 groups of health workers (women and men) involved in the implementation of the usual activities of the national neglected tropical diseases program.

A total of 72 interviews were conducted, including 66 semi-structured interviews and 6 focus groups.

#### 2.2.4. Data Collection

The semi-structured interviews and focus groups were conducted by two sociologists. The regional focal points for neglected tropical diseases facilitated the recruitment of participants and identified the interview locations. Data were collected using a guide developed by a multidisciplinary team. The guide was designed on the basis of literature data and the results of the quantitative study carried out in the Maritime region. The guide included two parts of 07 questions each to assess the acceptability and feasibility of the intervention. Questions on acceptability included the following: (1) the acceptability of index cases to disclose their status to family contacts; (2) the acceptability of index cases to disclose their status to neighboring contacts; (3) the acceptability of index cases to disclose their status to social contacts; (4) the acceptability of index cases to list all their contacts; (5) the acceptability of identified contacts to be screened; and (6) the acceptability of screened contacts to take SDR. Feasibility questions included the following: (1) contact listing by the health worker at the time of diagnosis; (2) contact tracing by the community health worker; (3) contact tracing at home; (4) contact tracing at a health center; (5) administration of SDR by the health worker; (6) follow-up of contacts by the community health worker; and (7) the management of adverse events by health workers. A transversal question was asked at the end of the guide to gather proposals for the implementation of this intervention (What would you suggest for the implementation of this intervention in Togo/in your community?). Individual interviews and group discussions took an average of 1 h 30 min, and additional notes were taken. The sessions were conducted in French and in a local language, where necessary, to ensure good understanding and ease of expression for the participants. The guides were translated into local languages and tested before being finalized.

#### 2.2.5. Data Processing and Analysis

All individual interviews and focus groups were recorded and transcribed for content analysis. Two main themes were selected for analysis: the acceptability and feasibility of implementing SDR-PEP for contacts of leprosy patients. All data were analyzed by meticulously reading the transcripts and classifying the data by previously defined themes (acceptability or feasibility). Similarities and differences in responses were identified within each group as well as between groups. Illustrative citations were extracted for each theme.

### 2.3. Ethical Considerations

The study was approved by Togo’s Bioethics Committee for Health Research (No 035/2021/CBRS dated 6 October 2021). Signed informed consent was obtained from each participant or his/her legal representative prior to the administration of the questionnaire. Anonymity was ensured by using a unique identification number for each respondent. A financial compensation of CFA 2000 (about EUR 3) was granted to each participant for travel expenses.

## 3. Results

### 3.1. Quantitative Results

#### 3.1.1. Socio-Demographic Profile of Index Leprosy Cases

In 2021, 24 leprosy cases were registered in four health districts (Avé, Vo, Yoto, and Zio) of the Maritime region. The participation rate of index cases in the study was 100%. The median age of index cases was 45 years (interquartile range: 34–65 years). In the Zio district, 83.3% of patients were not attending school. All index cases were multi-bacillary leprosy cases. The socio-demographic profile of index cases is presented in Table 1.

#### 3.1.2. Contact Enrolment

All 24 index cases provided a list of their contacts. Contacts of an index case who had not been traced by community health workers were not included. Thus, only the contacts of 23 index patients were included in the final analysis. A total of 235 contacts were included (an average of 10 contacts per index case). Thirty-eight (16.2%) contacts did not come for screening for various reasons (travel, sick relatives, unjustified reasons). A further 14 (5.9%) contacts were not eligible for SDR administration according to the exclusion criteria. Reasons for exclusion from the study included the following: children under 5 (n = 4), pregnancy (n = 3), signs of leprosy (n = 1), suspected liver or kidney disease (n = 4), and use of rifampicin in the last two years (n = 2). All other contacts received SDR. A total of 183 contacts received SDR and were followed up for 10 months.

#### 3.1.3. Socio-Demographic Profile of Contacts

The proportion of contacts varied from 6.56% in the Vo district to 48.08% in the Zio district. The median age of contacts was 33 years (interquartile range = 18–45 years). Nearly two-thirds (66.1%) of contacts were in school, with a higher proportion in the Vo (83.3%) and Zio (75%) districts (*p* = 0.016). Family contacts were the most numerous, accounting for 49.2% of all contacts (*p* = 0.002). The socio-demographic and clinical distribution of contacts is presented in Table 2.

#### 3.1.4. Follow-Up of Contacts

During follow-up, adverse events were reported during the first week after taking rifampicin. These included 173 cases (94.5%) of red urine, 4 cases (2.2%) of allergy (generalized pruritus), and one case (0.5%) of nausea and vomiting. No other adverse events were reported during the remainder of the follow-up.

### 3.2. Qualitative Results of the Study

A total of 72 interviews were carried out, including 66 semi-structured interviews and six focus groups. Two main themes were selected for analysis: the acceptability and feasibility of SDR-PEP for contacts of leprosy patients.

#### 3.2.1. Acceptability of SDR-PEP for Contacts of Leprosy Patients

Acceptability includes, on the one hand, the acceptance of index cases to disclose their status to contacts and to list their contacts (family, social, and neighborhood contacts) and, on the other hand, the acceptance of contacts to be screened and to take rifampicin.

##### Acceptability of Index Cases to Disclose Their Status to Contacts

Regarding to disclosure of the diagnosis to family and neighboring contacts, almost all the participants we met were in favor. An index case reported the following:

“With awareness, I understood that the disease is due to a microbe and I’m currently under treatment. As far as I’m concerned, I’m happy to tell my family that I’ve been diagnosed positive”.

(Index case)

But regarding social contacts, some feel that there could be reticence in this context:

“What’s certain in the immediate household is that there are no worries, but the problem is with social contacts”.

(Health district director)

##### Acceptability of Index Cases to List Their Contacts

Listing consists in helping the patient to establish a list of contact cases, suitable for enrolment. On the whole, the participants we met were unanimous in their belief that the indexes would adhere to this listing action. One leprosy patient reported the following:

“Yes, I’ll accept, so that the others too can be taken care of and protected”.

(Index case)

##### Contact Acceptability of Screening and Rifampicin Use

Contact acceptance of screening and rifampicin use was a major point of concern in this study. Almost all the contacts we met were in favor of screening. They reported the following:

“I’m going to accept it because it’s a disease. And we’ve been told that prevention is better than cure. If you detect the disease early and take precautions, it won’t lead to complications and you have a better chance of being cured. The way I know people, they’ll accept it”.

(Contact)

The final objective is for eligible contacts to take the SDR. The majority of respondents were positive about the acceptability of contacts taking the drug. The following are some of their comments:

“I’ll take it, especially as it’s free. If you had to pay for it, some people would say they didn’t have any money, but since it’s free, a lot of people will accept”.

(Contact)

“We’ll take the product because we need to protect ourselves”.

(Contacts)

#### 3.2.2. Feasibility of SDR-PEP for Contacts of Leprosy Patients

The feasibility of contact tracing, screening, and follow-up was assessed.

##### Feasibility of Contact Tracing by Community Health Workers

All those interviewed were in favor of contact tracing by community health workers. One community health worker reported the following:

“For tracing contact cases, those who are in the community, it would be easy”.

(community health workers)

##### Feasibility of Contact Tracing

An examination of the information collected reveals that, in all health regions, training of health professionals is an essential prerequisite for contact tracing. One health district director reported the following:

“In terms of human resources, I would say that we won’t have any problem screening contact cases if the staff are trained before the intervention”.

(Health district director)

During the interviews, the participants were asked to give their views on the feasibility of contact tracing locations. An examination of their responses reveals a divided position. Some people prefer screening to be carried out at home, while others do not, for various reasons:

“[...] Generally speaking, people living in very remote areas find it difficult to get to health facilities, due to a lack of means of transport. So, home screening would eliminate the problems associated with travel and the availability of people”.

(Health district director)

“Home screening would be better. Because if it’s at the center, people won’t come. But if we go to them in their homes, they’re more likely to come”.

(community health workers)

“I think it’s better to do screening at the hospital. There would be less stigma. If they come to the hospital, it would be like for any other disease”.

(Health district director)

##### Feasibility of Contact Follow-Up

Most participants were in favor of a follow-up of contact cases by community health workers under the supervision of health facility managers and NTD focal points after the administration of SDR. One participant stated the following:

“Community health workers are an integral part of the health system. They take part in a lot of activities, so it’s an activity they can also easily carry out”.

(NTD regional focal point)

Regarding the management of side effects after SDR administration, participants reported the following:

“The management of side effects must be made free of charge. If contacts are informed, this will encourage them. We also need to inform contacts about side effects in advance, and tell them to come to the health center as soon as these signs appear”.

(Health district director)

## 4. Discussion

For this quantitative and qualitative study, 24 index cases and 183 contact cases were enrolled and 72 interviews were conducted. The study described the safety, acceptability, and feasibility of SDR-PEP for contacts of leprosy patients in Togo.

### 4.1. Safety of SDR-PEP for Contacts of Leprosy Patients

Studies evaluating the safety of SDR-PEP are rare. In a review by Balqis et al., only two studies reported the safety of SDR-PEP for contacts of leprosy patients [12]. A cohort study by Richardus et al. involving 179,769 leprosy contacts reported one case of allergic reaction following SDR administration, and no serious adverse events were reported [13]. A systematic review by Ferreira et al. reported that adverse effects with SDR were absent or minimal in leprosy contacts, with reported adverse effects of cutaneous reaction such as flushing and itching 30 min after SDR intake [14]. In our study, during follow-up, red urine, allergy (generalized pruritus), and nausea and vomiting were reported during the first week after taking rifampicin. Adverse events reported during the first week following rifampicin intake were managed and then ameliorated a few days later. No serious adverse effects were reported during this first week, and no other adverse events were reported during the rest of the follow-up. SDR used as prophylaxis is generally an intervention that subjects tolerate well [8]. Nevertheless, the possibility of (rare) side effects and adverse events should be clearly explained to the subjects receiving it, and they should be monitored and followed up.

### 4.2. Acceptability of SDR-PEP for Contacts of Leprosy Patients

All the index cases identified agreed to take part in the study. This indicates that fear of revealing their status was not an obstacle. The same observation was also made during the individual interviews and focus groups, both by the patients: “As far as I’m concerned, I’m happy to tell my family that I’ve tested positive”. And other non-patient participants observed that “If I were in the patient’s shoes, I’d agree to tell them”. This high rate of acceptance of index cases has been observed in other studies, such as those carried out by Apte et al., where an acceptability rate of 99% was recorded [15]. However, a reluctance linked to the risk of stigmatization in the event of disclosure of their pathological condition to their neighbors and sometimes even to household members has been noted among index patients [16]. In our study, during interviews, some participants mentioned their concerns about disclosing patients’ status to social contacts: “In the immediate household, there are no worries, but the problem is with social contacts”. Indeed, this was observed during the quantitative survey, with 15.8% of social contacts being recruited. This may indirectly reflect the refusal of index cases to disclose their status to social contacts. However, the surveillance study conducted by Mounra et al. revealed that social contacts were at greater risk of developing leprosy [17]. To overcome these barriers, alternative approaches to contact tracing need to be explored. Community-wide screening, often referred to as general screening, is one option that can be adopted to achieve good contact recruitment coverage in highly endemic communities [13,16]. The type of contact screening (targeted and/or community screening) should be clearly defined before the deployment of this intervention in Togo.

No eligible contacts refused to take rifampicin in our study. The same observation was made by Feenstra et al., who reported that contacts were quite willing to take rifampicin [18]. In their study, Apte et al. reported an acceptability of 98.6% [15]. In our study, some contacts claimed rifampicin for family members who were not identified as contacts. This could be due to a misinterpretation that SDR totally protects against the development of leprosy, rather than reducing the risk of developing it. Awareness should be raised to correct this information before and during strategy implementation.

### 4.3. Feasibility of SDR-PEP for Contacts of Leprosy Patients

A total of 235 contacts were recorded among the 23 index patients, i.e., an average of 10 contacts per index case. Contact tracing and the administration of SDR are budget-intensive interventions, requiring more human and logistical resources. It is necessary to define in advance the target number of contacts per index patient with the expected benefits in terms of leprosy prevention and burden reduction [19]. The ideal number of contacts depends on the local epidemiological situation and the characteristics of the health system and logistical and/or financial resources.

The availability of contacts is also a factor to be taken into account when enrolling them. In our survey, 16.2% of contacts did not turn up on the day of screening because they were not in the locality or were busy with other activities. Seeking out contacts at certain times of day (school, office hours) or climatic seasons (planting and harvesting), or in communities with a high proportion of migrant workers, is associated with particular challenges linked to the reduced likelihood of meeting them at home [20]. In such situations, repeated visits may be necessary to capture the maximum number of contacts. Alternatively, contact tracing can be carried out as soon as possible after diagnosis, periodically in a given geographical area, annually as part of a special campaign, or a combination of these approaches [11]. The timing and frequency of contact tracing must be defined before the intervention is implemented.

Among the contacts examined, one case of leprosy was diagnosed and put on treatment according to the national protocol for the management of leprosy in Togo. This strategy not only reduces the risk of leprosy transmission among contacts of leprosy patients but also enables the early diagnosis of cases in the community and rapid treatment. This helps to contain the spread of infection and prevent disability [17].

### 4.4. Strengths and Limitations

This study of rifampicin prophylaxis of leprosy contacts was the first in Togo. To our knowledge, no study has been carried out to assess the acceptability and feasibility of this intervention prior to its implementation. One of the strengths of this study is the mixed survey that was carried out.

The quantitative study carried out in the Maritime region was of great importance. It enabled us to describe the acceptability and operational feasibility of this intervention in real-life situations.

Finally, although the subjects who took part in the interviews during the qualitative phase of the study were not chosen at random, we ensured that the main players were represented according to socio-cultural background, geographical distribution, and gender wherever possible. This approach enabled us to gather the perceptions of key stakeholders who might be involved in the implementation of single-dose post-exposure chemoprophylaxis with rifampicin in contact cases of leprosy in Togo.

## 5. Conclusions

This mixed-method study reveals that single-dose rifampicin as post-exposure prophylaxis for contacts of leprosy patients is safe and accepted by leprosy patients, their contacts, healthcare workers, and their community and is feasible in Togo. However, a number of conditions need to be considered for more effective results. These include the fight against stigmatization among the population and capacity-building for those involved, especially at the peripheral level, in diagnosis; the development of national guidelines on research, screening, and administration of the SDR to contacts of leprosy patients, including a one-week follow-up period for adverse events; and material and financial support for the national program in charge of leprosy control in Togo.

## Figures and Tables

**Table 1:** Socio-demographic and clinical characteristics of index patients by health district (N = 24).

	Health District				
	Avé (n = 8)	Vo (n = 3)	Yoto (n = 7)	Zio (n = 6)	Total (N = 24)
Age (years)					
Median [IQR]	48 [30–70]	41 [35–62]	39 [35–56]	56 [44–65]	45 [34–65]
Mean (SD)	49 (23)	51 (28)	44 (18)	54 (13)	49 (19)
Minimum–Maximum	22–80	29–82	18–70	37–65	18–82
Gender, n (%)					
Male	4 (50.0)	1 (33.3)	6 (85.7)	2 (33.3)	13 (54.2)
Female	4 (50.0)	2 (66.7)	1 (14.3)	4 (66.7)	11 (45.8)
Marital status, n (%)					
Living alone	2 (25.0)	2 (66.7)	2 (28.6)	3 (50.0)	9 (37.5)
Married	6 (75.0)	1 (33.3)	5 (71.4)	3 (50.0)	15 (62.5)
Level of education, n (%)					
No schooling	4 (50.0)	1 (33.3)	3 (42.9)	5 (83.3)	13 (54.2)
Educated	4 (50.0)	2 (66.7)	4 (57.1)	1 (16.7)	11 (45.8)
Occupation, n (%)					
Farmer	7 (87.5)	2 (66.7)	4 (57.1)	2 (33.3)	15 (62.5)
Other ***	1 (12.5)	1 (33.3)	3 (42.9)	4 (66.7)	9 (37.5)
Type of leprosy (MB), n (%)	8 (100.0)	3 (100.0)	7 (100.0)	6 (100.0)	24 (100.0)

*** Seamstress, Miller, Electrician, Bricklayer, Blacksmith, Plumber; Teacher; Student; Retailer. IQR = Interquartile Range; SD = Standard Deviation; MB = Multi-bacillary.

**Table 2:** Socio-demographic characteristics of contacts by health district (N = 183).

	Health District					*p*
	Ave (n = 40)	Vo (n = 12)	Yoto (n = 43)	Zio (n = 88)	Total (N = 183)	
Age (years)						
Median [IQR]	30 [12–46]	26 [15–56]	33 [20–42]	35 [21–46]	33 [18–45]	
Mean (SD)	30 (20)	32 (21)	34 (18)	35 (19)	34 (19)	
Minimum–Maximum	5–70	6–60	6–78	6–83	5–83	
Gender, n (%)						0.4
Male	23 (57.5)	5 (41.7)	17 (39.5)	46 (52.3)	91 (49.7)	
Female	17 (42.5)	7 (58.3)	26 (60.5)	42 (47.7)	92 (50.3)	
Marital status, n (%)						0.2
Living alone	19 (47.5)	8 (66.7)	14 (32.6)	35 (39.8)	76 (41.5)	
Married	21 (52.5)	4 (33.3)	29 (67.4)	53 (60.2)	107 (58.5)	
Level of education, n (%)						0.016
No schooling	17 (42.5)	2 (16.7)	21 (48.8)	22 (25.0)	62 (33.9)	
Educated	23 (57.5)	10 (83.3)	22 (51.2)	66 (75.0)	121 (66.1)	
Occupation, n (%)						0.085
Farmer	17 (42.5)	8 (66.7)	15 (34.9)	48 (54.5)	88 (48.1)	
Other ***	23 (57.5)	4 (33.3)	28 (65.1)	40 (45.5)	95 (51.9)	
Type of contact, n (%)						0.002
Family	25 (62.5)	11 (91.7)	22 (51.2)	32 (36.4)	90 (49.2)	
Neighbor	13 (32.5)	1 (8.3)	15 (34.9)	35 (39.8)	64 (35.0)	
Social	2 (5.0)	0 (0.0)	6 (14.0)	21 (23.9)	29 (15.8)	

*** Seamstress, Miller, Electrician, Bricklayer, Tinkerer, Blacksmith, Plumber; Teacher; Student; Housewife; Retailer. IQR = Interquartile Range; SD = Standard Deviation.

## Data Availability

The data presented in this study are available on request from the corresponding author.
